# Conciliation or Palliation?

**Published:** 1919-03-01

**Authors:** 


### THE MATRONS' AND SISTERS' DEPARTMENT.

### CONCILIATION OR PALLIATION?

THE proceedings at the Conference summoned by the National Council of Women last week to consider the question of nurses' hours have considerably puzzled a good many of our readers. Among the matrons who accepted the invitation to attend there was obviously no suspicion of the fact that they were assisting in an organised attempt to supersede the functions of the College of Nursing. Yet before they had left the room they had been insidiously drawn into assenting to the setting up of a new body to take over the work of considering and reporting upon the conditions of employment of nurses and the salaries paid to them which the College of Nursing is now actually engaged upon. We are greatly surprised that the President of the National Council should have sanctioned so grave a breach of etiquette to be perpetrated under the auspices of her society. We regret that either Miss Cox Davies or Miss Finch should have held out hopes that the Council of the College which they were informally representing would consent to abandon duties to which they had been called on the mandate of 12,000 nurses in order to delegate them to a committee formed haphazard at a poorly attended meeting. It is well known that many members of the College of Nursing have so great an abhorrence of strife that they are willing to go considerable lengths to meet the demands of the small hostile party which is at present blocking the road to complete unanimity in the nursing world. We have every sympathy with this ardent longing for peace. But the road to peace does not lie in joining forces with the railers. It can only be won by carrying out a straightforward and consistent policy. It is confidence in the inauguration of such a policy which is hourly bringing crowds of new members into the College, and the College authorities must look to their own members for support, must study the interests of nurses as a whole, develop their organisation on broad lines, and leave the malcontents alone. When the right time comes the malcontents will be only too thankful to join the majority, and no one will want to remember the past against them. While they still show themselves irreconcilable nothing is to be gained but much may be lost by making efforts to placate them.

Consider this matter of the conference. Matrons attended it in all good faith. Whom did they find themselves invited to meet? The lady who for the last three years has persistently attacked all the matrons on the College Council—all the matrons who are endeavouring to bring unity into the profession—as time-servers, as autocrats, as employers bent on sweating their staff, as pseudo-nurses not worthy to be reckoned in the fold, as disingenuous, endeavouring by tricks to gain an ascendency over all the nurses in the kingdom and use it for their own ends. In the speech which Mrs. Bedford Fenwick made on this occasion she could not refrain from introducing the King Charles's Head of her eloquence the iniquity of the lay managers. But there was no word about tyrannical matrons. As these matrons had been invited to take part in offering a direct insult to the College of Nursing they were regarded as belonging to the core of the profession. But it is impossible to have it both ways. Either matrons as a class are as evil-minded as the opponents of the College would have us believe—and in that case they are certainly not the people to be allowed to have any say in the matter of nurses' hours and nurses' pay—or they are what we all know them to be, women of exceptional ability who have reached their present position by virtue of hard work, women for the most part with wise heads and warm hearts willing to spend themselves for the honour of their profession. In that case an apology is due to them for the many improper words used of their exertions in establishing the College, and until that apology has been made by Mrs. Fenwick and the other traducers who follow her like sheep, attempts at conciliation merely confuse the issue.

The opponents of unity in the nursing profession are few in number. Probably there are not more than three or four who really count. But they are very astute and entirely incapable of being placated. To put the hand in theirs is instantly to be drawn into enmity with half the nursing world. We have seen no smallest sign of any desire on their part to conciliate or unite. The conference was an exceedingly clever and well-thought-out plan for stepping in between the College and its members, and presenting themselves before the eye of the public in the familiar guise of "Short's your friend, not Codlin." We deny the authority by means of which this committee which aims at superseding the functions of the College has come into existence. We deny its competence to investigate the question either of nurses' salaries or their hours. Such a committee to do any good must collect evidence from matrons all over the country. Are they to be asked to give information about their affairs to the lady who has branded them again and again as incompetent and insincere?

In our judgment matrons will be well advised quite placidly to let Mrs. Bedford Fenwick and her committee alone to talk about nurses' hours and salaries as long as they like. A committee appointed as this committee was is not entitled to the confidence, much less to the support, of trained nurses who know where their best interests lie and are united to maintain them.

